# Micro-Patterned Surfaces That Exploit Stigmergy to Inhibit Biofilm Expansion

**DOI:** 10.3389/fmicb.2016.02157

**Published:** 2017-01-23

**Authors:** Erin S. Gloag, Christopher Elbadawi, Cameron J. Zachreson, Igor Aharonovich, Milos Toth, Ian G. Charles, Lynne Turnbull, Cynthia B. Whitchurch

**Affiliations:** ^1^The ithree institute, University of Technology SydneyUltimo, NSW, Australia; ^2^School of Mathematical and Physical Sciences, University of Technology SydneyUltimo, NSW, Australia

**Keywords:** twitching motility, swarming motility, collective behavior, self-organization, biofilm, tfp, T4P

## Abstract

Twitching motility is a mode of surface translocation that is mediated by the extension and retraction of type IV pili and which, depending on the conditions, enables migration of individual cells or can manifest as a complex multicellular collective behavior that leads to biofilm expansion. When twitching motility occurs at the interface of an abiotic surface and solidified nutrient media, it can lead to the emergence of extensive self-organized patterns of interconnected trails that form as a consequence of the actively migrating bacteria forging a furrow network in the substratum beneath the expanding biofilm. These furrows appear to direct bacterial movements much in the same way that roads and footpaths coordinate motor vehicle and human pedestrian traffic. Self-organizing systems such as these can be accounted for by the concept of stigmergy which describes self-organization that emerges through indirect communication via persistent signals within the environment. Many bacterial communities are able to actively migrate across solid and semi-solid surfaces through complex multicellular collective behaviors such as twitching motility and flagella-mediated swarming motility. Here, we have examined the potential of exploiting the stigmergic behavior of furrow-mediated trail following as a means of controlling bacterial biofilm expansion along abiotic surfaces. We found that incorporation of a series of parallel micro-fabricated furrows significantly impeded active biofilm expansion by *Pseudomonas aeruginosa* and *Proteus vulgaris*. We observed that in both cases bacterial movements tended to be directed along the furrows. We also observed that narrow furrows were most effective at disrupting biofilm expansion as they impeded the ability of cells to self-organize into multicellular assemblies required for escape from the furrows and migration into new territory. Our results suggest that the implementation of micro-fabricated furrows that exploit stigmergy may be a novel approach to impeding active biofilm expansion across abiotic surfaces such as those used in medical and industrial settings.

## Introduction

Bacterial biofilms are communities of bacteria that are encased within a self-produced polymeric matrix. Bacteria in the environment predominately exist within biofilms and it is now recognized that many bacterial infections can be attributed to biofilms that are associated with elevated resistances to antibiotics and the immune system (Costerton et al., 1987; Hall-Stoodley et al., 2004). Whilst biofilms are often considered as sessile communities, under appropriate conditions the biofilms of many species of bacteria also have the ability to actively expand across surfaces via complex multi-cellular behaviors such as type IV pili-mediated twitching motility or flagella-mediated swarming motility (Harshey, 2003; Whitchurch, 2006; Kearns, 2010). These collective behaviors have been recognized as mechanisms of biofilm dispersal and have been implicated in the spread of infections along implanted medical devices such as occurs during catheter associated urinary tract infections (CAUTIs) (Costerton et al., 1987; Nickel et al., 1992; Inglis, 1993; Sabbuba et al., 2002; Hall-Stoodley et al., 2004).

CAUTIs are the most common cause of nosocomial infections, accounting for 40% of reported cases (Maki and Tambyah, 2001). The morbidity associated with CAUTIs develops once the ascending biofilm has reached the bladder and kidneys where it causes symptomatic infections (Nicolle, 2001). Biofilm expansion along urinary catheters has been associated with two main routes of entry by the infecting organism; intra- and extra-luminal (Inglis, 1993; Stoodley et al., 1999; Purevdorj et al., 2002; Sabbuba et al., 2002). The intra-luminal route accounts for ~1/3 of CAUTIs, where the biofilm ascends within the catheter lumen. The extra-luminal route accounts for the remaining 2/3, where the biofilm ascends at the interstitial space between the catheter surface and the urethra (Maki and Tambyah, 2001).

Twitching motility is a mode of surface translocation that is mediated by the extension and retraction of type IV pili and which, depending on the conditions, enables migration of individual cells or can manifest as a complex multicellular collective behavior that leads to biofilm expansion. When the CAUTI pathogen *Pseudomonas aeruginosa* is cultured at the interface between semi-solidified nutrient media and an abiotic surface, the resulting biofilms rapidly expand in this interstitial space via twitching motility (Semmler et al., 1999; Whitchurch, 2006). Under some conditions, twitching motility-mediated expansion of *P. aeruginosa* interstitial biofilms manifests characteristic micromorphological features including the assembly of rafts of cells at the leading edge which enable active migration into new territories, behind which forms an intricate, lattice-like network of trails comprised of motile cells (Semmler et al., 1999). In a recent attempt to understand how *P. aeruginosa* self-organizes into this dramatic trail network, we determined that during twitching motility-mediated biofilm expansion, *P. aeruginosa* cells forge furrows in the semi-solid substratum. These furrows guide subsequent bacterial cell movements, much in the same way that roads and footpaths coordinate motor vehicle and pedestrian traffic (Gloag et al., 2013a,b), which leads to the emergence of trails. Interestingly, under conditions that promote twitching motility by individual cells such as during the early stages of biofilm formation on glass submerged in liquid nutrient media, *P. aeruginosa* demonstrates trail-following behavior along trails comprised of the Psl exopolysaccharide (Zhao et al., 2013). Many self-organizing systems that utilize trail-following behaviors can be described by the concept of stigmergy, which is a universal phenomenon in which self-organization emerges from indirect communication through persistent signals within the environment (Grasse, 1959; Theraulaz and Bonabeau, 1999). We have recently proposed that stigmergy is an organizing principle that describes a range of bacterial collective behaviors (Gloag et al., 2013a, 2015). Here we have explored whether we could exploit stigmergic furrow-following behaviors of *P. aeruginosa* to inhibit active biofilm expansion across abiotic surfaces.

## Materials and methods

### Bacterial strains and media

Bacterial strains used in this study were the *P. aeruginosa* strain PAK and the *Proteus vulgaris* strain ATCC 13315. *P. aeruginosa* PAK was maintained on 1.5% LBA [10 g/L tryptone (Oxoid), 5 g/L yeast extract (Oxoid), 5 g/L NaCl, 1.5% agar (Oxoid)] and *Pr. vulgaris* ATCC 13315 was maintained on low salt 1.5% LBA (2.5 g/L NaCl) at 37°C. *P. aeruginosa* interstitial biofilms were cultured on nutrient media [4 g/L tryptone (Oxoid), 2 g/L yeast extract (Oxoid), 2 g/L NaCl, 1 g/L MgSO_4_.7H_2_O] solidified with 8 g/L gellan gum (MP Biomedicals; twitching motility gellan gum; TMGG). *Pr. vulgaris* interstitial biofilms were grown on LB solidified with 8 g/L gellan gum (Luria Bertani gellan gum; LBGG).

### Surface micro-fabrication

The micro-fabricated transverse furrow design consists of a series of lateral furrows repeating down the length of one half of a 22 × 40 mm surface, the other half being left smooth, i.e., without micro-fabricated features. This design was patterned to a photoresist coated silicon wafer (negative NLOF 2020 photoresist) using photolithography. Excess photoresist was removed by cleaning with O_2_ plasma. Aluminum of thicknesses 0.5 and 1 μm were deposited by thermal evaporation on the silicon wafer coated with the patterned photoresist furrows. The silicon wafer was then submerged in N-methly-2-pyrrlidone (NMP) at 80°C to remove the remaining photoresist and the accompanying aluminum deposits, leaving behind the furrow template as a negative mold on the silicon wafer (Supplementary Figure 1).

Positive molds were then cast into thin polydimethylsiloxane (PDMS) films, ~1 mm in height by mixing Sylgard 184 PDMS (Dow Corning) in a 1:10 ratio of curing agent to base and curing for 12 h at 65°C. Three PDMS films were made for each furrow parameter template. Furrow parameters will be described as width × depth × inter-furrow distance.

### Interstitial biofilm assay

**Figure 1:**
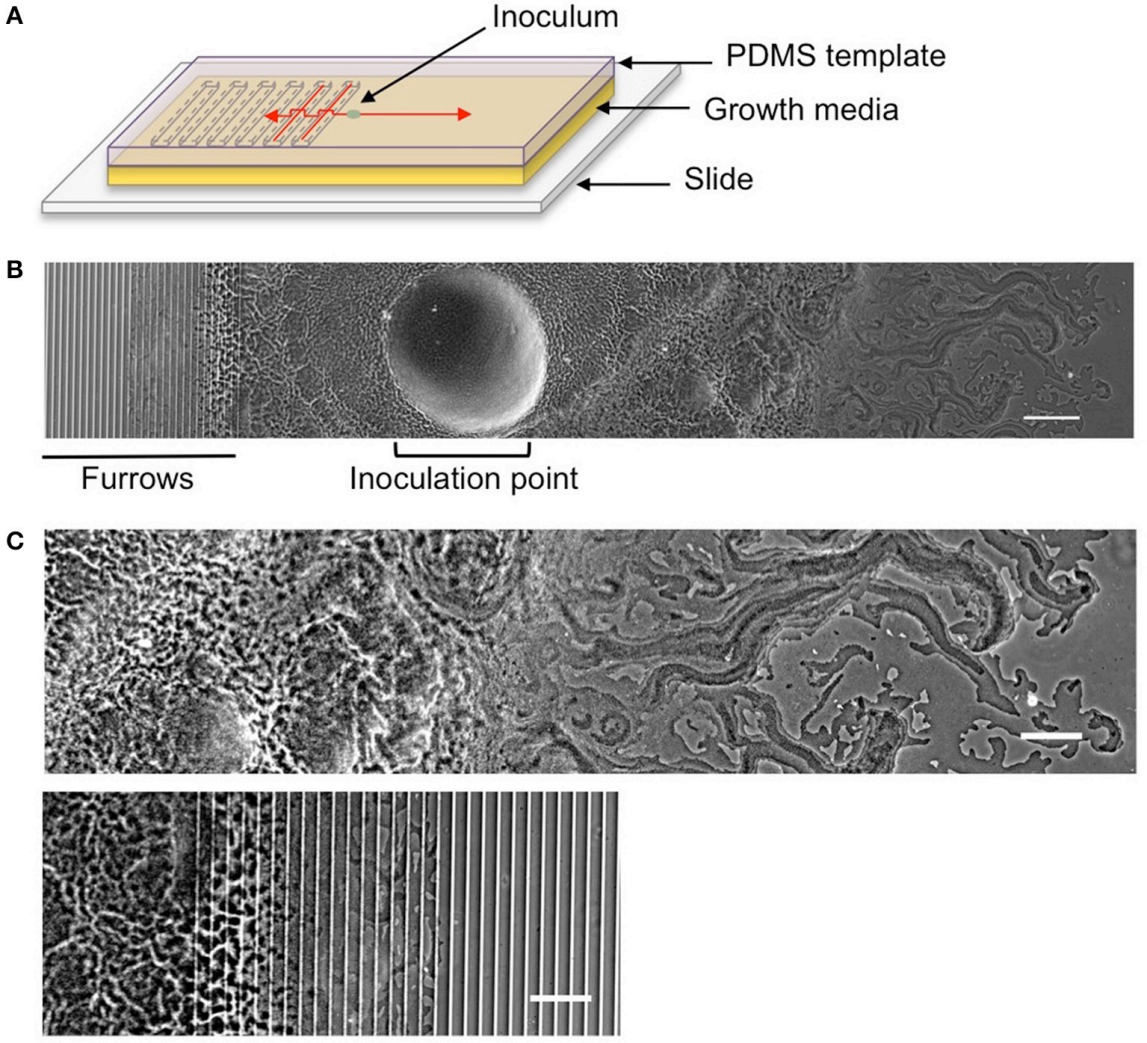
**The interstitial biofilm assay used to assess the extent of inhibition of biofilm expansion. (A)** Model of the interstitial biofilm assay. A PDMS template was fabricated that contained a series of transverse furrows along one half as shown. The PDMS was inoculated with a bacterial culture 1 mm away from the start of the repeating furrow design. This was then laid down on a glass slide coated in semi-solidified nutrient media, inoculation and furrow side down and incubated at 37°C. Red arrows indicate the direction of biofilm expansion across each side of the template. **(B)** Low magnification phase-contrast stitched image of a 4 h *P. aeruginosa* interstitial biofilm on a 5 × 1 × 20 μm furrow template. The biofilm is orientated as indicated in the cartoon above. The micro-fabricated furrows appear as phase-bright bands. Scale bar 200 μm. **(C)** The leading edges of the *P. aeruginosa* biofilm depicted in **(B)**. Upper panel is the leading edge on the smooth side and the lower panel is the leading edge on the furrow side. Both regions are cropped equidistant from the inoculation point and demonstrate the reduced biofilm expansion in the presence of furrows with dimensions of 5 μm (width) × 1 μm (depth) × 20 μm (inter-furrow spacing) compared to that on the smooth side. Scale bar 100 μm.

A modified interstitial biofilm assay (Turnbull and Whitchurch, 2014) was used as follows. The PDMS template was inoculated, rather than the nutrient media, by dotting a pipette tip coated in an inoculum from an overnight plate culture, 1 mm from the start of the micro-fabricated furrow design. This PDMS template was then used in replacement of the glass coverslip in the interstitial biofilm assay (Figure 1A). Interstitial biofilms of *P. aeruginosa* and *Pr. vulgaris* were grown at 37°C for 24 and 16 h, respectively. The 3 PDMS sets for each furrow parameter template were each repeated across 3 separate days (*n* = 9) for *P. aeruginosa* interstitial biofilms and 4 separate days (*n* = 12) for *Pr. vulgaris* interstitial biofilms, due to increased variability of the extent of *Pr. vulgaris* biofilm expansion compared to that of *P. aeruginosa*.

### Imaging interstitial biofilms

Low magnification phase-contrast microscopy images were taken across the length of the biofilm, starting at the leading edge of the biofilm within the micro-fabricated furrows right across to the leading edge on the opposite smooth side, making sure to include the point of inoculation. The slide was also scanned using an Epson Perfection V37 scanner (Epson) to obtain a macroscopic view of the biofilm. High magnification time-lapse microscopy was performed on each side of the biofilm across 30 min with a capture rate of one frame every 2 s to compare the biofilm expansion within the micro-fabricated furrows to that on the smooth side.

### Image analysis

The low magnification images were stitched within FIJI using the automated stitching plugin Grid/Collection stitching (Preibisch et al., 2009; Schindelin et al., 2012). The line tool in FIJI was used to measure the length from the inoculation point to the leading edge of the biofilm on both the furrows and smooth side. The distance from the inoculation point to the start of the furrows was also measured and this distanced subtracted from both biofilm lengths to account for any variation of this distance between experiments. These adjusted lengths were then used in subsequent analysis, unless specified. The expansion rate was calculated to account for these adjusted lengths and therefore represent the expansion rate across the time the leading edge was within the micro-fabricated furrows. To calculate the percentage reduction of interstitial biofilm expansion the difference in biofilm length on the smooth and furrow side was calculated and divided by the length of the biofilm on the smooth side. This was then expressed as a percentage by multiplying by 100.

Data are presented as mean ± *SD*. One-way ANOVA with a Tukey's *post-hoc* test and Student's *t*-test was used to compare data sets. Analyses were performed and data presented using GraphPad Prism v.6 (Graphpad Software). Statistical significance was determined using a *p* < 0.05.

## Results

### Rational for micro-patterned surface design

Micro-patterned surfaces were designed to exploit the stigmergic furrow-following behavior used by *P. aeruginosa* to self-organize during active biofilm expansion (Gloag et al., 2013a,b). Our aim was to determine if a series of parallel micro-fabricated furrows would be effective at inhibiting biofilm expansion. A series of transverse furrows were micro-fabricated on the surface of one half of a thin slab of PDMS, with the opposite half left smooth (Figures 1A,B). The dimensions of the micro-fabricated furrows were based on the dimensions of the furrows underlying *P. aeruginosa* biofilms (Gloag et al., 2013a,b). We have previously identified that furrows beneath the leading rafts and the lattice network were ~20 and 10 μm wide, respectively (Gloag et al., 2013a). Therefore, we generated micro-fabricated furrows with widths of 5, 10, 20, 50, and 100 μm to both mimic the dimensions of biofilm furrows and to identify an optimal range of inhibition. We had also determined that the depths of the biofilm furrows were on average 0.2 μm, with 0.5 μm the greatest depth measured (Gloag et al., 2013b). As we were attempting to maximize the confinement of cells within the micro-fabricated furrows during biofilm expansion we assayed 0.5 μm deep furrows to mimic the biofilm furrows as well as 1 μm depths to encompass the full height of a *P. aeruginosa* cell, which we have previously found to be an average height of 0.68 μm (Gloag et al., 2013b). Furrow dimensions deeper than 1 μm were not tested as we wanted to avoid cells becoming rapidly motile via swimming motility which was likely to occur in the deeper volume of liquid that would pool in the furrows. Therefore the furrow dimensions assayed were widths of 5, 10, 20, 50 and 100 μm, each at depths of 0.5 and 1 μm. Inter-furrow distances of 10, 15, and 20 μm were examined. The furrow dimensions are referred throughout the text as width × depth × inter-furrow distances.

### Micro-fabricated furrows impede *P. aeruginosa* active biofilm expansion

**Figure 2:**
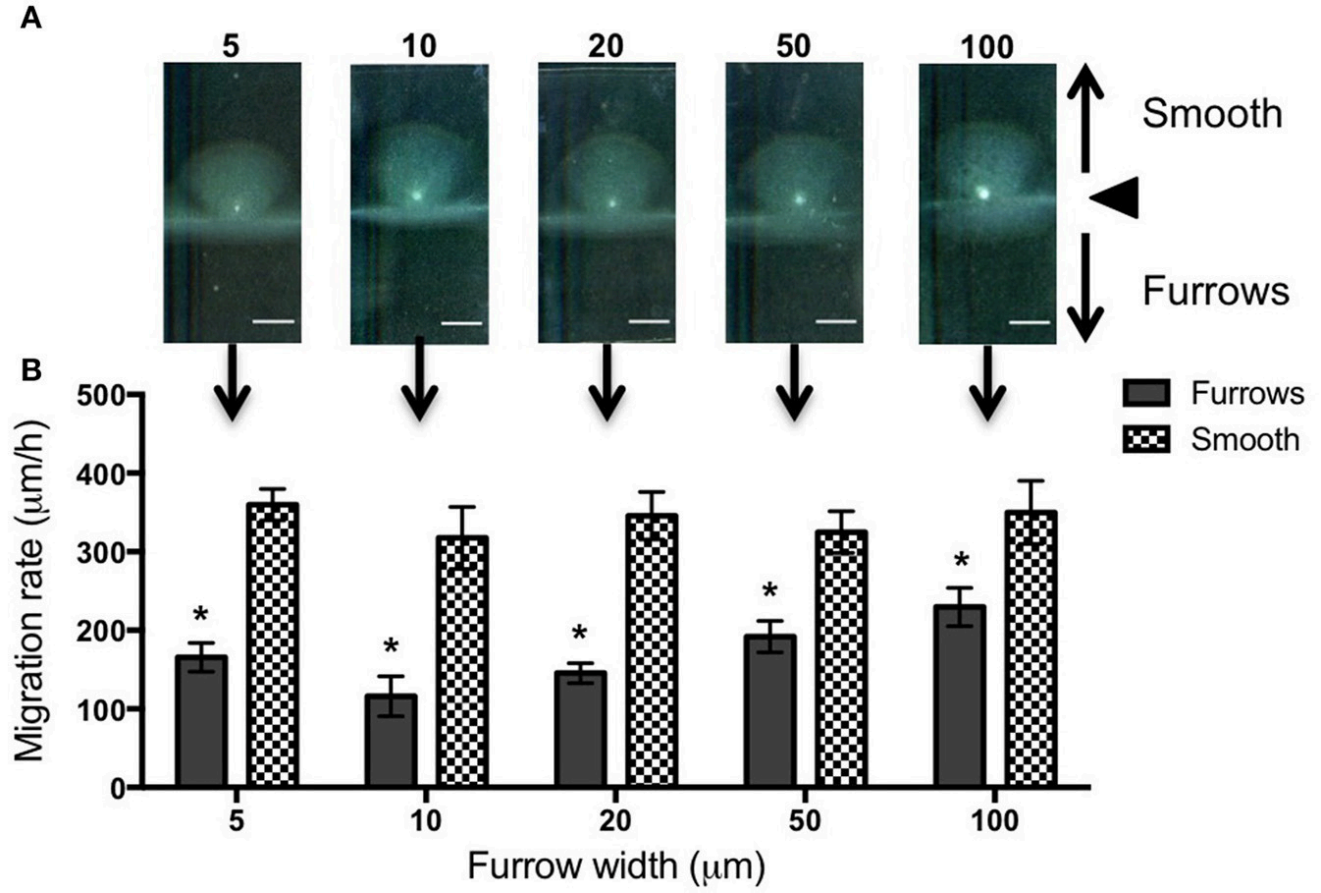
**Inhibition of ***P. aeruginosa*** interstitial biofilm expansion by 0.5 μm deep transverse micro-fabricated furrows. (A)** Macroscopic scans of *P. aeruginosa* biofilms after 24 h. The inoculation point (black triangle) is seen in the center as a bright white point. Biofilm migration direction is indicated by the black arrows. On the smooth side of the PDMS template above the inoculation point, the biofilm is seen as a semi-circular fan, and below the inoculation point on the micro-fabricated furrow side the biofilm is seen as a wide rectangle. Scale bar 5 mm. Furrow widths (μm) are indicated above the scan. All furrow designs had depths of 0.5 μm and inter-furrow distances of 20 μm. The furrow dimensions for each template correlate to that indicated in the graph below. **(B)** The net forward migration rate of *P. aeruginosa* interstitial biofilms across both the furrow and smooth side of each PDMS template. The furrow widths are indicated on the *x*-axis. All furrow designs had depths of 0.5 μm and inter-furrow distances of 20 μm. (Depicted is mean ± *SD, n* = 9). ^*^p < 0.0001.

**Figure 3:**
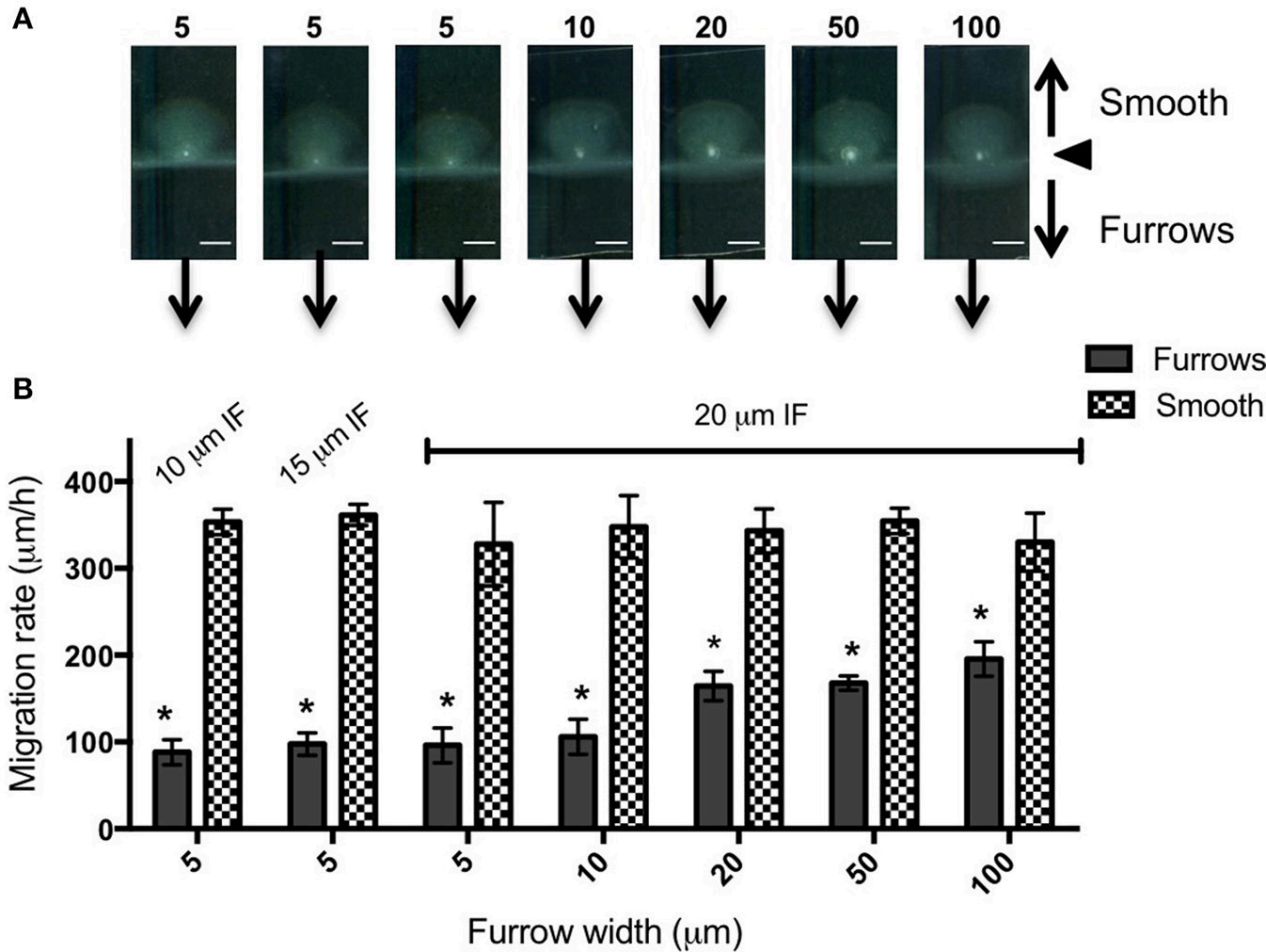
**Inhibition of ***P. aeruginosa*** interstitial biofilm expansion by 1 μm deep transverse micro-fabricated furrows (A)** Macroscopic scans of *P. aeruginosa* biofilms after 24 h. To the top is the smooth side of the PDMS template and to the bottom is the micro-fabricated furrow side. The white point in the center is the inoculation point. Scale bar 5 mm. The furrow dimensions for each template correlate to that indicated in the graph below. Furrow widths (μm) are indicated above the scan. All furrow designs had depths of 1 μm. Inter-furrow (IF) distances are indicated in **(B)**. **(B)** The net forward migration rate of *P. aeruginosa* interstitial biofilms across both the furrow and smooth side of each PDMS template. The furrow widths are indicated on the x-axis. All furrow designs had depths of 1 μm. Inter-furrow (IF) distances (μm) are indicated above the relevant data. Depicted is mean ± *SD, n* = 9. ^*^p < 0.0001.

To test the effects of different furrow dimensions on *P. aeruginosa* biofilm expansion we modified our interstitial biofilm assay in which the biofilm is cultured at the interstitial space between solidified nutrient media and a glass coverslip (Turnbull and Whitchurch, 2014), by replacing the glass coverslip with a thin slab of micro-patterned PDMS (Figures 1A,B). We initially utilized an inter-furrow distance of 20 μm with the furrow depth and width varying between the micro-patterned PDMS templates. We found that all micro-patterned surfaces tested caused a reduction in the net expansion of *P. aeruginosa* biofilms relative to the unpatterned control. We observed extensive expansion from the inoculation point on the smooth side and comparatively limited expansion in the presence of the micro-fabricated furrows (Figures 1B,C). Macroscopically, *P. aeruginosa* interstitial biofilms were observed on the smooth side as an extensive semi-circle radiating outward from the inoculation point (Figures 2A, 3A), whereas on the furrow side the biofilms were thin and narrow, extending across the width of the PDMS template (Figures 2A, 3A). This was reflected in a significant reduction in the net expansion rate of *P. aeruginosa* biofilms across the micro-fabricated furrows, compared to the smooth side, for all furrow parameters tested (Figures 2B, 3B; *p* < 0.0001). For the 1 μm deep furrows a decrease in furrow width correlated to a greater reduction in biofilm expansion, that was observed macroscopically as a narrower biofilm in the presence of the furrows (Figure 3A; *p* < 0.0001). This trend was not apparent for the 0.5 μm deep furrows, where the inhibition of the 5 μm wide furrows was similar to that of the 20 and 50 μm wide furrows (Figure 2B). Furthermore, for the 5 μm wide furrows, the 1 μm depth inhibited biofilm expansion to a significantly greater extent compared to the furrows of 0.5 μm depth (71.0 ± 2.7 vs. 54.0 ± 3.6%, respectively; Figures 2B, 3B; *p* < 0.0001). For the other furrow widths tested there was a similar reduction at the two depths, with the exception of the 50 μm wide furrows, which showed a greater reduction with the 1 μm depth compared to 0.5 μm (*p* < 0.0001). Together these observations indicated that the 1 μm deep micro-fabricated furrows were more effective than 0.5 μm depth furrows at inhibiting biofilm expansion, presumably due to this depth encompassing the full height of the cells and confining the cell movements more effectively. As such, all subsequent experiments used 1 μm deep furrows.

In an attempt to further increase the inhibition of biofilm expansion over that observed for the 5 μm wide furrows, inter-furrow distances of 10 and 15 μm were also examined (Figure 3). While the 5 × 1 × 10 μm furrows (width × depth × inter-furrow spacing) showed the greatest reduction to *P. aeruginosa* biofilm expansion (75.0 ± 4.3%; Figure 3B), which was also observed macroscopically (Figure 3A), statistically the different inter-furrow distances had no effect, with the reduction being relatively similar across the different furrow dimensions (5 × 1 × 10 μm: 75.0 ± 4.3%, 5 × 1 × 15 μm: 73.0 ± 3.4%, and 5 × 1 × 20 μm: 71.0 ± 2.7%, respectively; Figure 3B). The reduction on *P. aeruginosa* biofilm expansion by the 5 × 1 × 10 μm furrows was significant in comparison to that of the 10 × 1 × 20 μm furrows (Figure 3B; *p* < 0.05), and overall the reductions where similar for the 5 and 10 μm wide furrows, with these displaying a significant reduction in comparison to furrows with widths of 20, 50, or 100 μm (Figure 3B; *p* < 0.0001). These observations indicate that a micro-patterned PDMS surface containing a series of parallel furrows is an effective means of reducing the overall rate of *P. aeruginosa* biofilm expansion.

To observe the interaction of *P. aeruginosa* cells with the micro-patterned surfaces during biofilm expansion, we used time-lapse phase-contrast microscopy to observe cell movements as they migrated across either smooth PDMS or PDMS micro-patterned with a series of 5 × 1 × 10 μm furrows (Supplementary Video 1). The *P. aeruginosa* interstitial biofilm on the smooth side migrated rapidly across the field of view (Supplementary Video 1). In contrast, in the presence of the furrows, progression of the *P. aeruginosa* biofilm was significantly impeded with cells largely confined to the micro-fabricated furrows (Supplementary Video 1).

Our analyses have also revealed that in order to escape the furrow and traverse across the inter-furrow regions, *P. aeruginosa* cells at the leading edge re-orient and organize into vanguard rafts (Supplementary Video 1). This process is similar to what has been previously observed during twitching motility-mediated biofilm expansion of *P. aeruginosa* where it has been noted that leading-edge raft assemblages are required for migration into new territories (Semmler et al., 1999; Gloag et al., 2013b).

**Figure 4:**
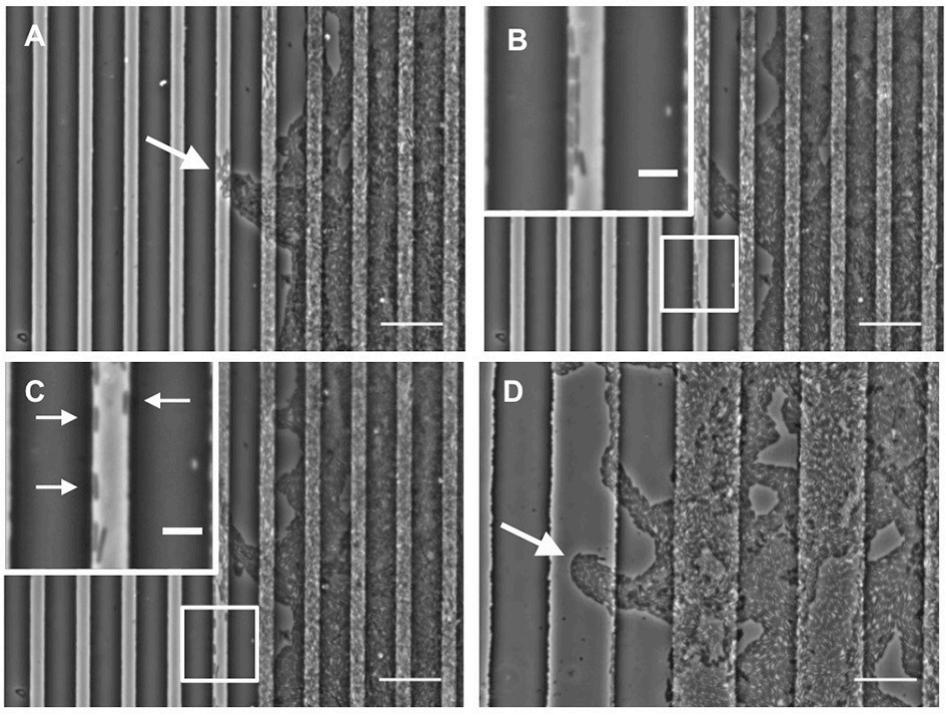
**Furrow edges guide ***P. aeruginosa*** movements**. Individual frames from Supplementary Video 1 showing *P. aeruginosa* cells interacting with the furrow edges during migration along furrows with dimensions of 5 μm (width) × 1 μm (depth) × 10 μm (inter-furrow spacing). **(A)** Leading raft formation is disrupted when entering 5 × 1 × 10 μm furrow (4.20 min, Supplementary Video 1). **(B)** Cells migrating along the edge of the furrow (14.20 min, Supplementary Video 1). **(C)** Cells displaying independent motility when interacting with the furrow edge. Arrows indicate cells that display single cell motility (19.40 min, Supplementary Video 1). **(D)** Intact leading raft within a furrow with dimensions of 20 μm (width) × 1 μm (depth) × 20 μm (inter-furrow spacing). Arrow indicates a leading raft assembly within the furrow that remains intact within the wider furrow. Scale bar 20 μm. Inset scale bar 5 μm.

Our time-series of *P. aeruginosa* twitching motility across micro-patterned PDMS also showed that as these leading edge rafts entered narrow furrows with widths of 5 or 10 μm, which showed the greatest inhibition of biofilm expansion, the aggregates of cells immediately disassembled and the cells altered their direction of motion to migrate along the length of the furrow (Figure 4A and Supplementary Video 1). These cells also appeared to preferentially migrate along the furrow edge, rather than migrating along the middle of the channel (Figure 4B and Supplementary Video 1). Furthermore, these cells appeared to migrate independently of neighboring cells, in some cases migrating as well isolated single cells (Figure 4C and Supplementary Video 1). Individual cell motility is rarely observed during twitching motility-mediated interstitial biofilm expansion of *P. aeruginosa* (Semmler et al., 1999). The observation that individual *P. aeruginosa* cells preferentially migrate along the furrow edge is reminiscent of the behavior previously reported for *Neisseria gonorrhoeae* diplococci which were observed to migrate via twitching motility along the edges of the channels etched in PDMS surfaces (Meel et al., 2012). It was proposed that the channel walls provided additional surfaces for the type IV pili of the *N. gonorrhoeae* to bind to facilitate twitching motility (Meel et al., 2012). Similarly, the type IV pili of *P. aeruginosa* may bind to the PDMS furrow edges in our micro-fabricated system, thereby enabling their individual movements and accounting for the preferential alignment of cells along these borders (Figure 4 and Supplementary Video 1).

In contrast, when observing biofilm expansion in the presence of the wider micro-fabricated furrows, we noted that for furrows with widths 20 μm or greater, the leading raft assemblages were maintained after entering the furrow (Figure 4D). As these wider furrows were less inhibitory to overall biofilm expansion (Figures 2, 3) we predict that the maintenance of these leading raft assemblages within the wider furrows facilitates a more rapid escape from the furrow, accounting for the greater net expansion across the PDMS surfaces micro-patterned with wider furrows (Figure 3B). In contrast, within the narrower furrows of 5 or 10 μm wide in which the leading rafts dispersed upon entering the furrow, the cells need to re-coordinate their collective behaviors in order to successfully re-organize into the aggregates required to escape from the furrow and traverse into new territory. It appears therefore that the narrower (5 and 10 μm) furrows are more efficient at disrupting bacterial collective behaviors causing the aggregates to disperse and guide the individual cells to migrate along the longitudinal direction of the furrow.

### Micro-fabricated furrows impede *Proteus vulgaris* active biofilm expansion

**Figure 5:**
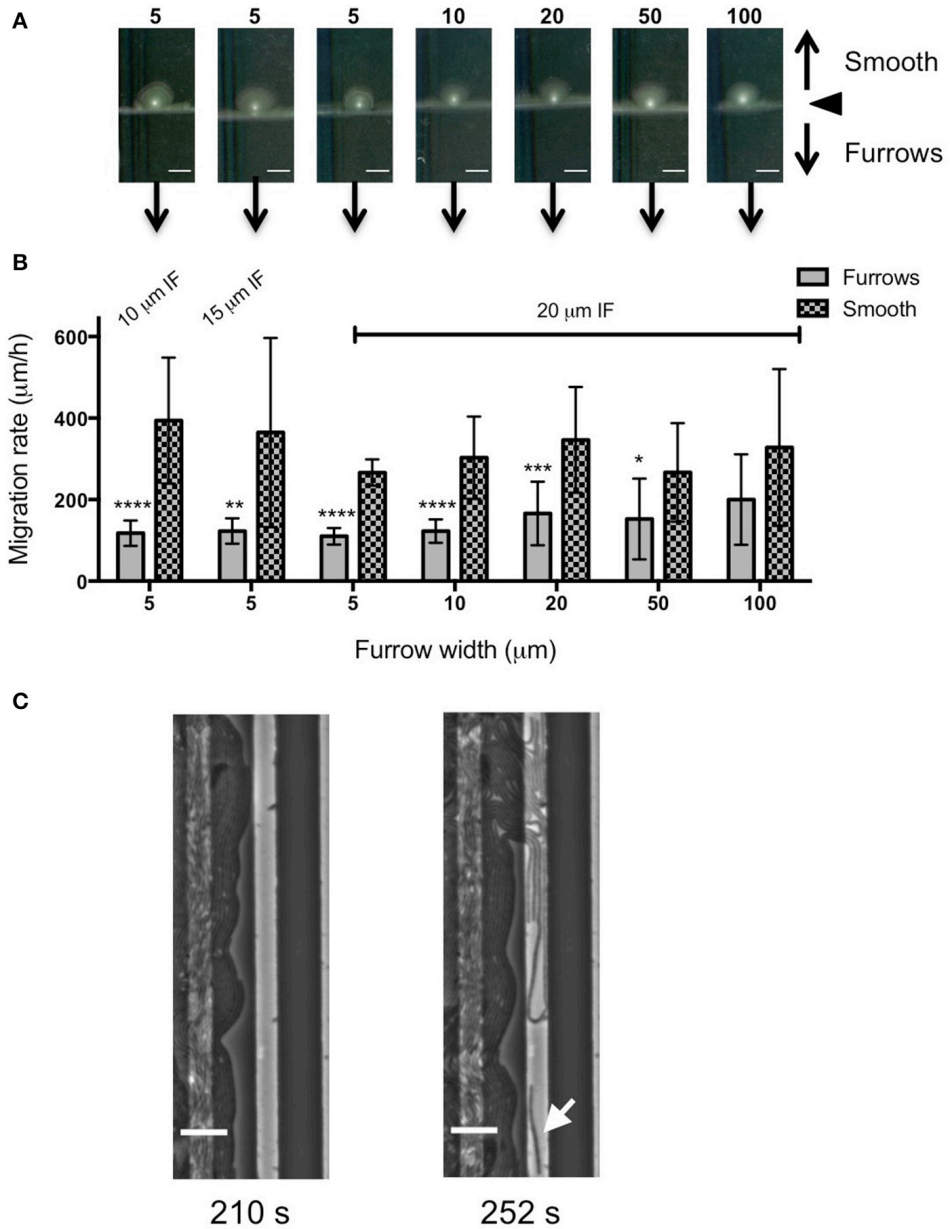
**Inhibition of ***Pr. vulgaris*** interstitial biofilm expansion by transverse micro-fabricated furrows (A)** Macroscopic scans of *Pr. vulgaris* biofilms after 16 h. To the top is the smooth side of the PDMS template and to the bottom is the micro-fabricated furrow side. The white point in the center is the inoculation point. Scale bar 5 mm. The furrow dimensions for each template correlate to that indicated in the graph below. Furrow widths (μm) are indicated above the scan. All furrow designs had depths of 1 μm. Inter-furrow (IF) distances are indicated in **(B)**. **(B)** The net forward migration rate of *Pr. vulgaris* interstitial biofilms across both the furrow and smooth side of each PDMS template. The furrow widths are indicated on the *x*-axis. All furrow designs had depths of 1 μm. Inter-furrow (IF) distances (μm) are indicated above the relevant data. Depicted is mean ± *SD, n* = 12. ^****^p < 0.0001,^***^p < 0.001, ^**^p < 0.01, ^*^p < 0.05. **(C)** Individual frames from Supplementary Video 3 showing *Pr. vulgaris* cells interacting with furrows with dimensions of 5 μm (width) × 1 μm (depth) × 10 μm (inter-furrow spacing) showing the swarm front being disrupted when entering the furrow. Arrow indicates a cell showing single cell behavior. Time of each frame (Supplementary Video 3) is denoted below in seconds. Scale bar 10 μm.

**Figure 6:**
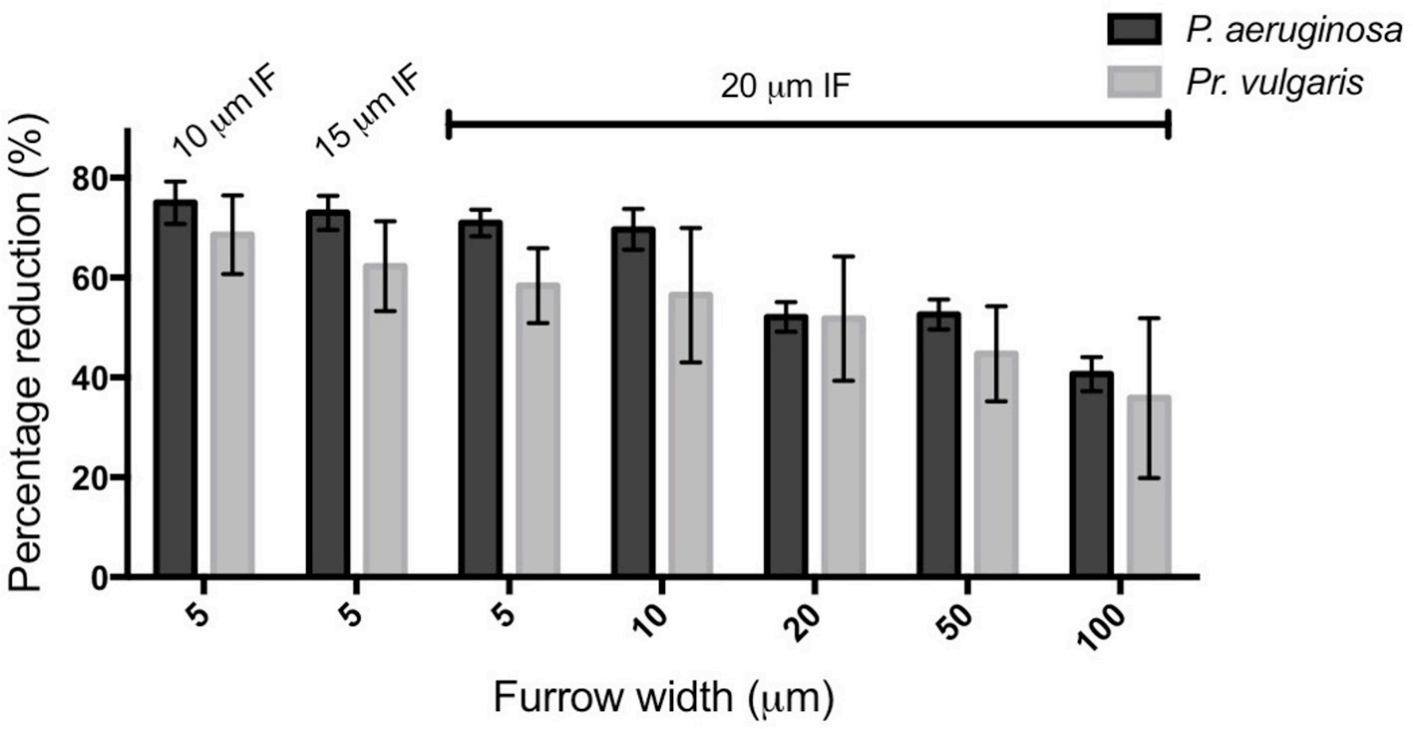
**Transverse micro-fabricated furrows impede the active expansion of ***P. aeruginosa*** and ***Pr. vulgaris*** interstitial biofilms**. The percentage reduction of *P. aeruginosa* and *Pr. vulgaris* biofilm expansion caused by each micro-fabricated furrow dimension when compared to the expansion across the corresponding smooth side. The furrow widths are indicated on the *x*-axis. All furrow designs had depths of 1 μm. Inter-furrow (IF) distances (μm) are indicated above the relevant data. Depicted is mean ± *SD, n* = 12 for *Pr. vulgaris* and *n* = 9 for *P. aeruginosa*.

We also examined the ability of 1 μm deep micro-fabricated furrows to inhibit biofilm expansion by *Pr. vulgaris*, another important CAUTI pathogen whose biofilms actively expand along catheter surfaces using flagella-mediated swarming motility (Fluit et al., 2001). We found that, similar to *P. aeruginosa*, in the presence of the micro-fabricated furrows the interstitial biofilms of *Pr. vulgaris* were narrow and extended across the width of the template when viewed macroscopically. This was in contrast to the biofilms cultured on the smooth side where they were observed as a semi-circle displaying a concentric circle pattern (Figure 5A). The concentric circle patterning is typical of the swarming motility of *Proteus* spp. and forms as a result of continuous rounds of cellular differentiation into filamentous hyper-flagellated swarmer cells, which mediate surface colonization via swarming motility, followed by consolidation via differentiation back into the normal rod cell, which are largely non-motile (Harshey, 1994; Rauprich et al., 1996). Due to these continuous rounds of differentiation cycles, there was variation in the measured expansion rates of the *Pr. vulgaris* biofilms, particularly on the smooth side (Figure 5B) depending on whether the cells had entered a motility or consolidation cycle by the end of the time course. We found that wider furrows were not as effective at controlling the collective behavior of *Pr. vulgaris* during biofilm expansion (Figure 5B) and as was observed for *P. aeruginosa*, narrow furrow widths correlated with a greater reduction to biofilm expansion (Figure 5B; *p* < 0.0001 for 5 × 1 × 10, 5 × 1 × 20, and 10 × 1 × 20 μm; *p* < 0.01 for 5 × 1 × 15 μm), with the 5 μm wide furrows displaying the greatest reduction (68.6 ± 7.9%; Figure 6).

To observe the interaction of *Pr. vulgaris* cells within the micro-fabricated furrows, phase-contrast time-lapse microscopy was performed using PDMS patterned with 5 × 1 × 10 μm furrow series (Supplementary Videos 2, 3). On the smooth side it was observed that the biofilm migrated rapidly via swarming motility. However, in the presence of the micro-fabricated furrows the biofilm advancement was impeded. We noted that as the leading front of the *Pr. vulgaris* swarm entered the furrow, the cells appeared to dissociate from the swarm front and change their direction of motion to migrate along the length of the furrow (Figure 5C and Supplementary Videos 2, 3). It therefore appears that the micro-patterned surface disrupts the cohesive swarm front and subsequently controls and guides the movements of individual cells to migrate along the furrows.

## Discussion

We have found that a series of micro-fabricated transverse furrows are effective at inhibiting the active biofilm expansion of two common CAUTI pathogens, *P. aeruginosa* and *Pr. vulgaris*. Furrow dimensions between 5–10 μm wide, 1 μm deep, and 10–20 μm inter-furrow distance were the most effective, as these reduced biofilm expansion of *P. aeruginosa* and *Pr. vulgaris* by up to ~75 and 70%, respectively (Figure 6). The reduction in biofilm expansion was found to be due the micro-patterned surfaces exploiting the tendency of bacteria to follow trails to coordinate their collective motion. For *P. aeruginosa* the micro-patterned surface specifically exploits the furrow following behavior used by this organism to self-organize during active biofilm expansion (Gloag et al., 2013a,b). By exploiting this stigmergic natural behavior the micro-fabricated furrows confined the migration of the cells, thereby controlling bacterial traffic during active biofilm expansion.

We found that the 5 and 10 μm wide furrows were the most successful at inhibiting biofilm expansion by *P. aeruginosa* (Figure 2C; *p* < 0.0001) and *Pr. vulgaris* (Figure 5B; *p* < 0.0001 for 5 × 1 × 10, 5 × 1 × 20, and 10 × 1 × 20 μm; *p* < 0.01 for 5 × 1 × 15 μm). These widths mimic the dimensions of furrows underlying *P. aeruginosa* interstitial biofilms that stigmergically coordinate cellular migration leading to the formation of interconnected trails (Gloag et al., 2013a,b). The formation of raft assemblages are essential for *P. aeruginosa* biofilm expansion into new territories (Semmler et al., 1999; Gloag et al., 2013b). These vanguard rafts are typically wide structures of ~20 μm (Gloag et al., 2013a) and the assembly of new rafts to break out from the side of a furrow and migrate into new territory is a highly orchestrated event (Gloag et al., 2013b). In this study, we found that micro-fabricated furrows with narrow dimensions (5 and 10 μm widths) prevented *P. aeruginosa* cells from self-organizing into the raft assemblages required to escape the furrow. Multilayer stacking of cells in the furrow enabled them to eventually traverse the furrow edge either by lifting or compressing the gellan gum, assemble into rafts and migrate across the interstitial space until they encountered the next furrow. The ability of narrow furrows to disassemble rafts, guide bacterial migration along the furrows, and inhibit assembly of new rafts results in significant inhibition of twitching motility-mediated biofilm expansion by *P. aeruginosa*.

We found that micro-patterned PDMS with narrow width dimensions were also effective at inhibiting swarming motility-mediated biofilm expansion by *Pr. vulgaris*. During the swarming motility of *Proteus* spp. the swarm front is highly cohesive, with the flagella from neighboring cells interweaving with one another forming extensive cellular interactions that coordinate their collective movements (Jones et al., 2004). However, we found that upon entering narrow furrows, *Pr. vulgaris* cells appeared to dissociate from the swarm front and migrate individually along the furrow. We suggest that these frequent disruptions to the swarm front and cells preferentially migrating along the furrows accounts for the inhibition of *Pr. vulgaris* interstitial biofilm expansion by micro-fabricated furrows.

There has been significant interest in the application of micro-patterned surfaces to prevent biofouling, with various surface topographies identified that reduce the attachment of bacteria, algal spores, and barnacle cyprids (Schumacher et al., 2007, 2008; Ling et al., 2014). More recently these surfaces have also been found to reduce the attachment and surface colonization of important human pathogens and thus their application to the surface of implanted and indwelling medical devices is now receiving increased attention (Chung et al., 2007; Reddy et al., 2011; Ivanova et al., 2012, 2013; Hasan et al., 2013; May et al., 2014). However, to our knowledge this is the first report of a micro-patterned surface design that aims to impede active biofilm expansion by exploiting stigmergic regulation of bacterial collective behaviors.

In conclusion here we have identified that furrow dimensions between 5–10 μm wide and 1 μm deep were the most effective at impeding the active biofilm expansion of two common nosocomial pathogens, *P. aeruginosa* and *Pr. vulgaris* by up to 75 and 70%, respectively. Our results suggest that exploiting bacterial stigmergic self-organization to control bacterial surface migration may be an effective non-antibiotic approach to prevent the incidence of device-associated infections.

## Author contributions

CW, LT, and IC conceived the project. EG performed the experiments. CE, CZ, IA, and MT produced the photoresist templates and PDMS molds. CW, EG, and LT analyzed the data and interpreted the results. EG and CW wrote the manuscript. All authors were involved in discussions of experimental design and have read and approved the manuscript.

## Funding

CW was supported by an Australian National Health and Medical Research Council Senior Research Fellowship (571905).

### Conflict of interest statement

The authors declare that the research was conducted in the absence of any commercial or financial relationships that could be construed as a potential conflict of interest.

## Supplementary material

Supplementary Figure 1 **Schematic of surface micro-fabrication method**. A clean silicon wafer **(A)** was coated in negative NLOF 2020 photoresist **(B)**. Prebake of the photoresist at 110°C for 1 min was performed to enhance adhesion to the substrate and minimize mask contamination **(C)**. A brightfield chrome on glass photomask was then aligned in the desired position over the photoresist and substrate **(D)** and exposed to UV radiation **(E)** to cross-link of exposed photoresist. The substrate then underwent a post bake again at 110°C for 1 min **(F)** to further cross-link and harden the exposed photoresist. The substrate was then immersed in AZ300 MIF developer **(G)** for 30 s to remove the unexposed photoresist leaving behind the patterned substrate. Aluminum was then thermally evaporated onto the substrate at the desired thickness **(H)** and liftoff was performed by immersing the substrate in N-methly-2-pyrrlidone (NMP) at 80°C to remove the remaining photoresist and the accompanying aluminum deposits **(I)** leaving behind the desired pattern with specified thickness on the silicon substrate. **(J)** PDMS was then poured over the negative mold and cured for 12 h at 65°C. PDMS film was peeled off leaving the photolithography defined pattern in the PDMS.

Supplementary Video 1 *****P. aeruginosa*** cells are preferentially confined to the micro-fabricated furrows during interstitial biofilm expansion**. Time-lapse phase-contrast microscopy was performed across 2000 s with a capture rate of one frame every 2 s to observe *P. aeruginosa* interstitial biofilm expansion across PDMS with micro-fabricated furrows with dimensions of 5 μm (width) × 1 μm (depth) × 10 μm (inter-furrow spacing). The left panel depicts the leading edge of *P. aeruginosa* biofilm migrating across the furrow side where the phase-bright bands are the furrows. Right panel is the leading edge migrating across the corresponding smooth side. Time is indicated in the top right hand corner. Scale bar 20 μm.

Supplementary Video 2 *****Pr. vulgaris*** cells are preferentially confined to the micro-fabricated furrows during interstitial biofilm expansion**. Time-lapse phase-contrast microscopy was performed across 2000 s with a capture rate of one frame every 2 s to observe *Pr. vulgaris* interstitial biofilm expansion across PDMS with micro-fabricated furrows with dimensions of 5 μm (width) × 1 μm (depth) × 10 μm (inter-furrow spacing). The left panel depicts the leading edge of *Pr. vulgaris* biofilm migrating across the furrow side where the phase-bright bands are the furrows. Right panel is the leading edge migrating across the corresponding smooth side. Time is indicated in the top right hand corner. Scale bar 20 μm.

Supplementary Video 3 **Micro-fabricated furrows disrupt ***Pr. vulgaris*** swarm front during interstitial biofilm expansion**. Time-lapse phase-contrast microscopy was performed across 500 s with a capture rate of one frame every 2 s to observe *Pr. vulgaris* interstitial biofilm expansion across PDMS with micro-fabricated furrows with dimensions of 5 μm (width) × 1 μm (depth) × 10 μm (inter-furrow spacing). Time is indicated in the top right hand corner. Scale bar 20 μm.
