# Supplementary figures and images for: Micro-Patterned Surfaces That Exploit Stigmergy to Inhibit Biofilm Expansion
Source: Front Microbiol. 2017 Jan 23;7:2157. doi: 10.3389/fmicb.2016.02157 (PMC5253354; doi:10.3389/fmicb.2016.02157)

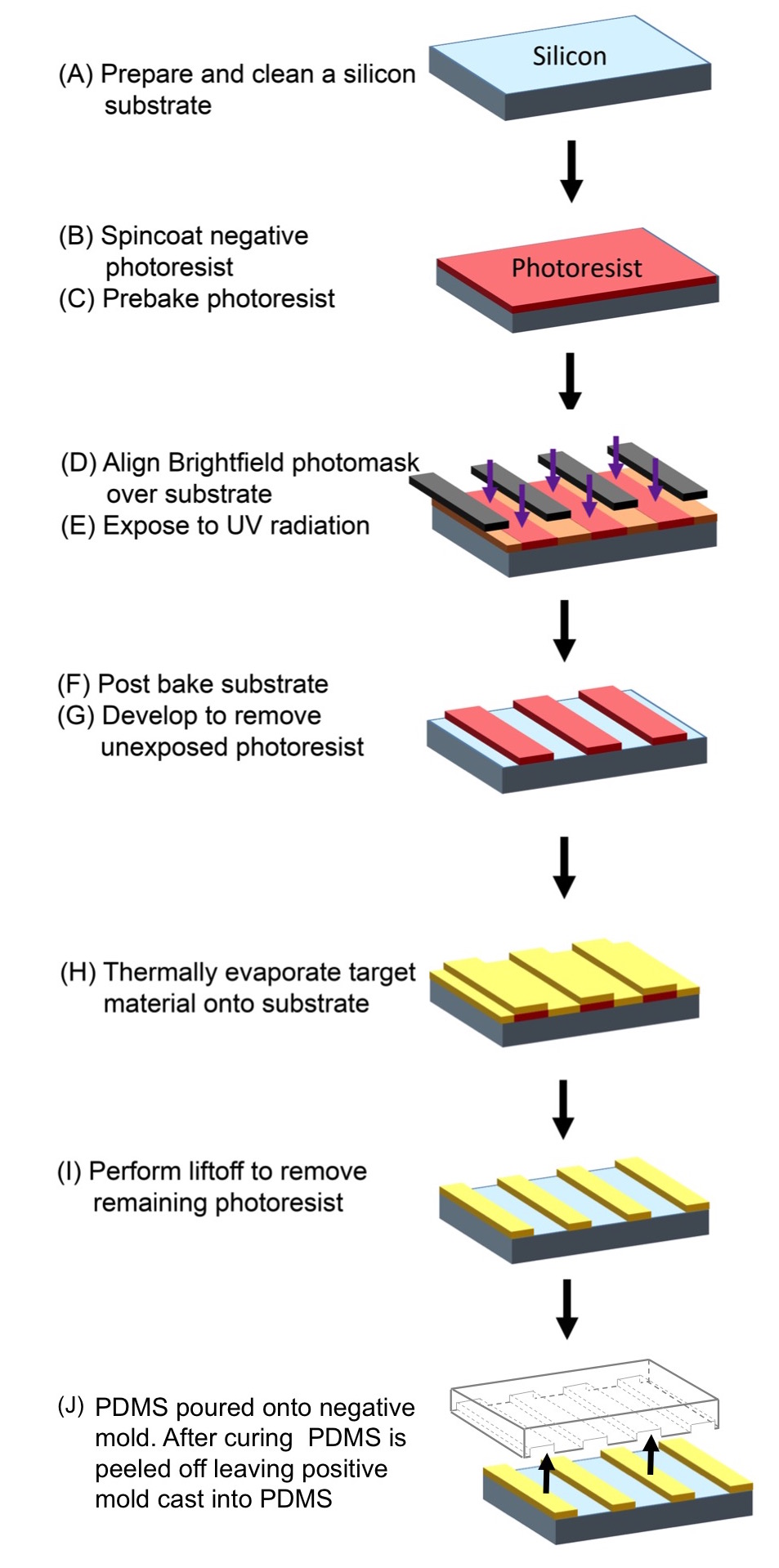

Supplement: Supplementary Figure 1 — Schematic of surface micro-fabrication method. A clean silicon wafer (A) was coated in negative NLOF 2020 photoresist (B). Prebake of the photoresist at 110°C for 1 min was performed to enhance adhesion to the substrate and minimize mask contamination (C). A brightfield chrome on glass photomask was then aligned in the desired position over the photoresist and substrate (D) and exposed to UV radiation (E) to cross-link of exposed photoresist. The substrate then underwent a post bake again at 110°C for 1 min (F) to further cross-link and harden the exposed photoresist. The substrate was then immersed in AZ300 MIF developer (G) for 30 s to remove the unexposed photoresist leaving behind the patterned substrate. Aluminum was then thermally evaporated onto the substrate at the desired thickness (H) and liftoff was performed by immersing the substrate in N-methly-2-pyrrlidone (NMP) at 80°C to remove the remaining photoresist and the accompanying aluminum deposits (I) leaving behind the desired pattern with specified thickness on the silicon substrate. (J) PDMS was then poured over the negative mold and cured for 12 h at 65°C. PDMS film was peeled off leaving the photolithography defined pattern in the PDMS. [file Image1.JPEG]
